# Effects of Non-Nutritive Sweeteners on Energy Intake, Body Weight and Postprandial Glycemia in Healthy and with Altered Glycemic Response Rats

**DOI:** 10.3390/foods10050958

**Published:** 2021-04-28

**Authors:** Meztli Ramos-García, Jorge Luis Ble-Castillo, Carlos García-Vázquez, Carlos Alfonso Tovilla-Zárate, Isela Esther Juárez-Rojop, Viridiana Olvera-Hernández, Alma Delia Genis-Mendoza, Rubén Córdova-Uscanga, Carlos Alfonso Álvarez-González, Juan Cuauhtémoc Díaz-Zagoya

**Affiliations:** 1 Centro de Investigación, División Académica de Ciencias de la Salud (DACS), Universidad Juárez Autónoma de Tabasco (UJAT), Villahermosa 86150, Mexico; meztli.garcia@hotmail.com (M.R.-G.); gbasecs@hotmail.com (C.G.-V.); iselajuarezrojop@hotmail.com (I.E.J.-R.); viryolvera11@gmail.com (V.O.-H.); cordova.1@live.com.mx (R.C.-U.); 2 División Académica Multidisciplinaria de Comalcalco, UJAT, Comalcalco 86650, Mexico; alfonso_tovillaz@yahoo.com.mx; 3 Laboratorio de Genómica de Enfermedades Psiquiátricas y Neurodegenerativas, Instituto Nacional de Medicina Genómica (INMEGEN), Ciudad de Mexico 14610, Mexico; adgenis@inmegen.gob.mx; 4 División Académica de Ciencias Biológicas (Dacbiol), UJAT, Villahermosa 86150, Mexico; alvarez_alfonso@hotmail.com; 5 División de Investigación, Facultad de Medicina, Universidad Nacional Autónoma de Mexico (UNAM), Ciudad de Mexico 04360, Mexico; zagoya@unam.mx

**Keywords:** appetite, aspartame, energy metabolism, stevia, sucralose, rebaudioside A

## Abstract

The aim of this study was to evaluate the effects of non-nutritive sweeteners (NNS) consumption on energy intake, body weight and postprandial glycemia in healthy and with altered glycemic response rats. Animals on normal diet (ND) or high-fat diet (HFD) were divided to receive NNS (sucralose, aspartame, stevia, rebaudioside A) or nutritive sweeteners (glucose, sucrose) for 8 weeks. The NNS were administered at doses equivalent to the human acceptable daily intake (ADI). A test using rapidly digestible starch was performed before and after treatments to estimate glycemic response. No effects of NNS consumption were observed on energy intake or body weight. Sucrose provoked an increased fluid consumption, however, energy intake, and weight gain were not altered. In ND, no effects of NNS on glycemic response were observed. In HFD, the glycemic response was increased after sucralose and stevia when only the final tolerance test was considered, however, after including the baseline test, these results were no longer significant compared to glucose. These findings provide further evidence suggesting that at the recommended doses, NNS do not alter feeding behavior, body weight or glycemic tolerance in healthy and with altered glycemic rats.

## 1. Introduction

The overconsumption of energy-dense foods, rich in sugar, has contributed to the rapid increase of metabolic disorders [1]. Non-nutritive sweeteners (NNS) are food additives with the advantage of providing high potency in sweetness but with few or no caloric content [2]. Worldwide NNS consumption has increased and Mexico exhibits a high proportion of nutritional products containing NNS [3]. The three most popular NNS are sucralose, aspartame and stevia; the first two being from synthetic origin (artificial) and the latter from natural sources [4,5].

Although NNS were developed to help control caloric intake, body weight, or glycemia concentrations in people with high risk for developing type 2 diabetes (T2DM) or already with diabetes [6], evidence has revealed that these additives may disturb glucose homeostasis and feeding behavior in animal models and humans [7]. Some of the proposed mechanisms to explain how these additives can provoke these alterations include the degradation of the ability to induce reward response in the central nervous system compared to sugar-sweetened foods or beverages, the alteration of incretin and insulin secretion, the upregulation of pro-inflammatory and adipogenesis promoting pathways and the gut microbiota disturbance [8,9].

The results regarding the effects of NNS on caloric consumption and weight gain have been mixed. While some indicate NNS may be beneficial [10,11], others report body weight gain [12]. In this line, the effects of NNS on glycemic response are also controversial. Some studies have found harmful effects. Pepino et al. found that sucralose induced an increase in glycemic and insulin responses in people with obesity [13]. Suez et al. showed that NNS induced glucose intolerance in rodents and humans [14]. In a more recent study, Romo-Romo et al. showed that a moderate consumption of sucralose (15% of ADI) for only 14 days induced a reduction on insulin sensitivity in healthy subjects [15]. These studies are in contrast with others who have reported no effects. Higgins et al. found that high doses of aspartame for 12 weeks, did not affect glycemia and body weight in lean adults [16]. A different study showed that the consumption of carbonated beverages with aspartame and ace-k for 12 weeks had no effect on insulin sensitivity in non-diabetic subjects [17].

Animal models have been used to investigate NNS effects because of the advantages of better control in the administered doses and a shorter treatment period. It is known that many foods of daily use for humans have a high NNS content which makes difficult to regulate the amount ingested. In addition, a common limitation of previous studies is the use of high NNS doses; in some cases, far beyond the currently established FDA-approved acceptable daily intake (ADI) in humans which may reduce the relevance of their findings [18,19]. Here, we used NNS doses from natural and artificial sources adjusted by daily fluid and body weight to be equivalent to the ADI. Although these doses could seem low for animal models, we consider them to be adequate for mimicking real-life human conditions. For comparison, the FDA estimates the ADI equivalents to be 75 tabletop sweetener packets (TSP) or 18–19 cans (1 can = 12 oz, or 355 mL) of diet cola for aspartame, 23 TSP or 6 cans of diet cola for sucralose and 9 TSP for stevia [20,21], which typically surpass the daily ordinary consumption in the general population. Regarding the period of intervention, the 8 weeks of NNS treatment chosen for the animals in the present study correspond to approximately 4.7 years of life in humans [22].

On the other hand, the conclusions about the effects on glycemic response have been usually based only on outcomes from an oral glucose tolerance test (OGTT) performed at the end of the intervention period administering oral pure glucose to the animals. Here, the glycemic responses were evaluated by an oral starch tolerance test (OSTT) carried out both at the end and at the beginning of the experiment. This latter test was performed administering rapidly digestible starch which provides quick rises in postprandial glycemia and could offer lower variability compared to the OGTT [23,24]. The baseline tolerance test allowed us to determine whether the post-test adjusted for pre-test scores differed between treatments. We hypothesized that the chronic consumption of NNS at doses equivalent to ADI would lead to an increased energy intake, weight gain and impaired glucose tolerance in healthy and with altered glycemic response rats.

## 2. Materials and Methods

### 2.1. Experimental Animals

Adult male Wistar rats weighing 150–200 g (6 to 8 weeks old) were provided by the Faculty of Medicine of the National Autonomous University of Mexico (UNAM) and housed in polycarbonate cages (four per cage) at controlled temperature (18 °C to 26 °C), ventilation cycles (12 to 15 air changes per hour), relative humidity (45% to 60%), and 12/12 h light/dark cycles (7:00 h to 19:00 h). The experimental procedures were managed according to the Official Mexican Standard NOM-062-ZOO-1999 and the guidelines established by the Research Committee for the Care and Use of Laboratory Animals of the UNAM. The study protocol was approved by the Investigation Committee of the Academic Division of Health Sciences, Juarez Autonomous University of Tabasco (Approval No. 002/CIP/DACS).

### 2.2. Diets

Normal diet (ND) consisted in standardized LabDiet Rodent^®^ 5001 chow (28.67% protein, 57.94% carbohydrate, 13.38% fat; 3.36 kcal/g), and pure water. High-fat diet (HFD) was daily prepared mixing standard diet with lard in pellets (4.6 kcal/g of energy, 26.7% carbohydrates, 13.2% proteins and 60.0% fat). High caloric diet (HCD) consisted of high-fat diet pellets (HFD) along with a 30% sucrose solution (1.2 kcal/mL). Previously to each study phase, animals underwent a one-week acclimation period receiving ND and pure water, except for the SUC30-treated rats which remained on 30% sucrose solution during the second acclimation period. All animals had access to food and fluid ad libitum during the experimental period.

### 2.3. Sweeteners

Commercially available sweeteners were used; sucralose from Sweeny^®^ plus (1.3% sucralose, 98.7% glucose), aspartame from Selecto Brand^®^ (4% aspartame, 96% glucose), and stevia from Selecto Brand^®^ (3% stevia, 97% glucose). Rebaudioside A (reb A) was purchased from Anhui Minmetals, Hefei, China (98% purity). Glucose from Roquette corporation^®^ (≥91% purity) and sucrose from Zulka^®^ (≥98% purity) were used. They were dissolved in pure water and presented to rats at room temperature.

### 2.4. Study Design

#### 2.4.1. Phase 1: Altered Glycemia Induction 

**Figure 1:**
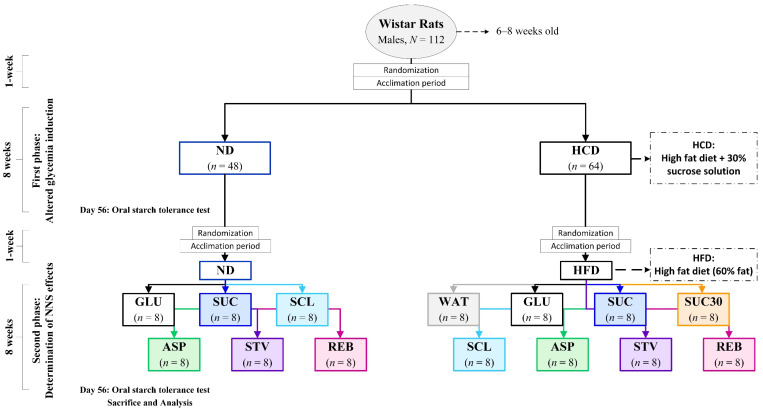
Schematic diagram of the experimental design.

One hundred and twelve male Wistar rats were divided into two dietary groups: one was given ND (*n* = 48) and the other one was supplied with HCD (*n* = 64) for 8 weeks (Figure 1). At the end of this period (day 56), an oral starch tolerance test (OSTT) was performed to all the animals to estimate the glycemic response.

#### 2.4.2. Phase 2: Determination of NNS Effects

On day 58, animals were randomly assigned to receive the different NNS treatments (Figure 1). ND was subdivided into six groups (*n* = 8) to receive for 8 weeks: 4% glucose (GLU), 4% sucrose (SUC), sucralose (5 mg/kg body weight (BW)/d) (SCL), aspartame (50 mg/kg BW/d) (ASP), stevia (4 mg/kg BW/d) (STV), and reb A (4 mg/kg BW/d) (REB). The HFD-fed rats were subdivided into eight groups; in addition of GLU, SUC, SCL, ASP, STV and REB mentioned above for ND, two more groups were considered: 30% sucrose (SUC30) and pure water (WAT) for 8 weeks. All the NNS treatments were administered in drinking water.

The administered doses of commercial NNS were equivalent to the ADI [25]. The concentration was adjusted daily to provide the desired dose to each animal based on the average of daily fluid consumption per day and BW using an Excel fact sheet. For the majority of the experimental period, the required doses were reached, except for the first two weeks where a modest variation was observed (see Supplementary Material, Figure S1). To control the high glucose content in the commercial sweeteners, a 4% glucose solution (GLU) was introduced as a primary control group. Glucose concentrations were adjusted to 4% in all the NNS groups (SCL, ASP, STV, REB). The quantity of pure glucose added to each NNS solution was daily calculated using an Excel fact sheet according to the next formula: Added glucose in g = [(40 g of glucose) − (glucose contained in NNS expressed in g)] / 1 L of solution. This procedure allowed maintaining the same glucose concentration per mL despite changes in daily NNS consumption. The SUC, SUC30 and WAT groups served as secondary comparators. All animals were assigned to the different treatments by stratified randomization using a computer-based online random number generator (www.random.org, accessed on 5 February 2021) according to the baseline body weights.

### 2.5. Measurements of Food Consumption, Total Energy Intake and Body Weight

In both ND and HCD in the first phase or HFD-fed rats in the second phase, the control of food intake and fluid consumption was conducted daily by subtracting the remaining amount from the supplied one. All the drinking solutions were prepared daily throughout the study. Moreover, data from NNS fluid consumption were captured per day/cage and adjusted to the desired dose (1 ADI) per day, using an Excel fact sheet. The rats were weighed twice a week at the same time in the morning using an electronic precision balance (Precision BJ 2200C). Weekly energy intake was calculated by the sum of consumed calories from pellets and fluid per cage with four animals, adjusted by the total BW of the animals and expressed as kcal/week/100 g BW per group, as previously has been reported [26,27].

### 2.6. Oral Starch Tolerance Test (OSTT)

Before and after NNS treatments, OSTTs were performed in all the animals to estimate the glycemic response. After 12 h of fasting, rats received a dose of 3 g/kg BW of Amioca^®^ (100% rapidly digestible starch; Ingredion, Mexico S.A. de C.V., Ags, Mexico) dissolved in 2 mL of water and administered by gavage. Blood samples were obtained via the tail vein puncture, before (time 0) and 30, 60, 90 and 120 min after starch administration for glucose determination using a FreeStyle Optium Neo glucometer from Abbott Laboratories.

### 2.7. Biochemical Measurements

At the end of the 8-week treatment period, animals were fasted overnight and anesthetized with a low dose of sodium pentobarbital (35 mg/kg/BW, i.p, PiSA^®^). Blood samples were collected via cardiac puncture through the chest wall. Serum was separated in duplicate and preserved at −70 °C for further analysis. The determinations of glucose, triglycerides, cholesterol, and HDL- cholesterol were performed using the A25 Clinical Chemistry Autoanalyzer System (BioSystems^®^ Reagents & Instruments S.A, Antioquia, Colombia). Plasma insulin levels were determined using a rat/mouse insulin ELISA kit from Millipore (EZRMI-13K, St. Louis, MO, USA) according to the instructions of the manufacturer. Insulin resistance was estimated according to the Homeostasis Model Assessment (HOMA-IR) which was calculated by the product of the fasting concentrations of glucose (mg/dL) and insulin (μU/mL) divided by 405 [28].

### 2.8. Statistical Analysis

The D’Agostino-Pearson normality test was performed to assess if the data exhibited a Gaussian distribution. Data on body weight evolution, and total energy intake through time during the first phase were analyzed by two-way repeated measures (RM) ANOVA and Sidak’s multiple comparisons test.

Weekly measures of fluid intake, food consumption, energy intake and body weight during the second phase (NNS treatments) were also analyzed by two-way RM ANOVA or mixed-effects model and Dunnett post hoc test, considering treatment as the between-group factor and time as the within-group factor. Data from total weight gain during NNS treatment were compared using a one-factor ANOVA and Dunnett’s post hoc test. To estimate differences between treatments from the final OSTT curve a one-way ANOVA and a Dunnett’s post hoc test was performed using the incremental glycemia area under curve (iAUC) values from glycemia changes from baseline (∆ glycemia). However, a posterior ANCOVA analysis was performed to investigate if the differences between treatments remained after controlling for the baseline glycemia iAUC values. Differences were considered statistically significant at *p* < 0.05. Data were processed and analyzed using GraphPad Prism Software version 7.0 (San Diego, CA, USA) or IBM SPSS Statistics version 26.0 (Armonk, NY, USA).

## 3. Results

### 3.1. Phase 1: Altered Glycemic Response Induction 

**Figure 2:**
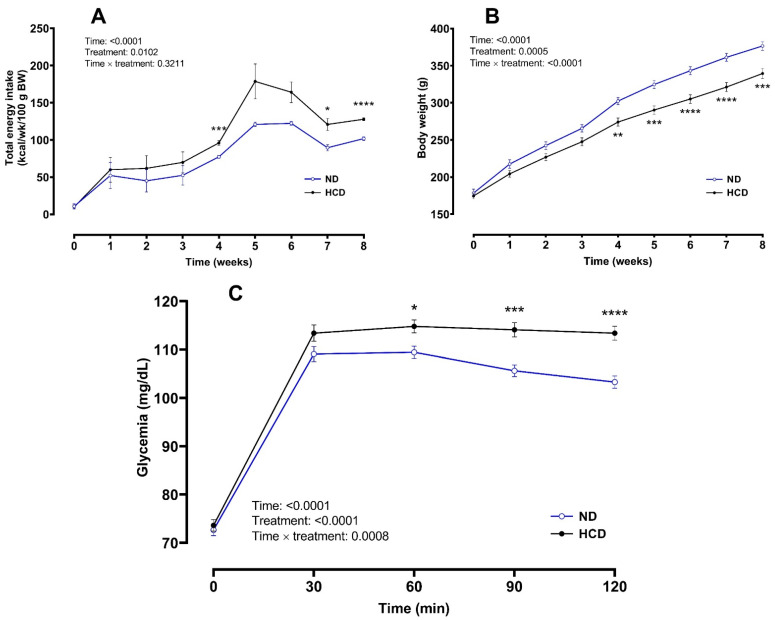
Total energy intake dynamics (**A**) and body weight evolution (**B**) over eight weeks on normal diet (ND) or high-caloric diet (HCD), and glycemic profiles (**C**) during an oral starch tolerance test (OSTT) at the end of the interventions (phase 1). Data are expressed as mean ± SEM. ND (*n* = 50) and HCD (*n* = 68), Two-way RM ANOVA followed by Sidak’s post hoc test was used. * *p* < 0.05, ** *p* < 0.01, *** *p* < 0.001, **** *p* < 0.0001.

All the animals presented similar body weight at the beginning of the treatment. The food intake was lower in the HCD group compared to the ND (50.77 ± 1.12 vs. 90.33 ± 1.55 g/kg BW/d, *p* < 0.0001). Similarly, the fluid intake was lower in the HCD group (98.95 ± 4.10 vs. 206.1 ± 7.42 mL/kg BW/d, *p* < 0.0001). Despite the lower consumption, HCD exhibited a higher total energy intake compared to the ND (*F* = 7.902, *p* = 0.0102 for treatment; *F* = 1.168, *p* = 0.3211 for time × treatment interaction and *F* = 31.25, *p* < 0.0001 for time factor) (Figure 2A), yet the weekly body weight evolution in the HCD was lower than the ND (*F* = 12.79, *p* = 0.0005 for treatment, *F* = 14.19, *p* < 0.0001 for time × treatment interaction and *F* = 1154, *p* < 0.0001 for time) (Figure 2B). At the end of week 8, the final body weight was lower in the HCD than in the ND (339.4 ± 6.806 g vs. 376.5 ± 5.712 g, *p* < 0.001). The glycemic response was higher after HCD than after ND (*F* = 17.76, *p* < 0.0001 for treatment factor; *F* = 4.862, *p* < 0.0001 for time × treatment interaction and *F* = 422.8, *p* < 0.0001 for time factor) (Figure 2C).

### 3.2. Phase 2: Effects of NNS 

#### 3.2.1. Effects of NNS on Energy Intake and Body Weight

**Figure 3:**
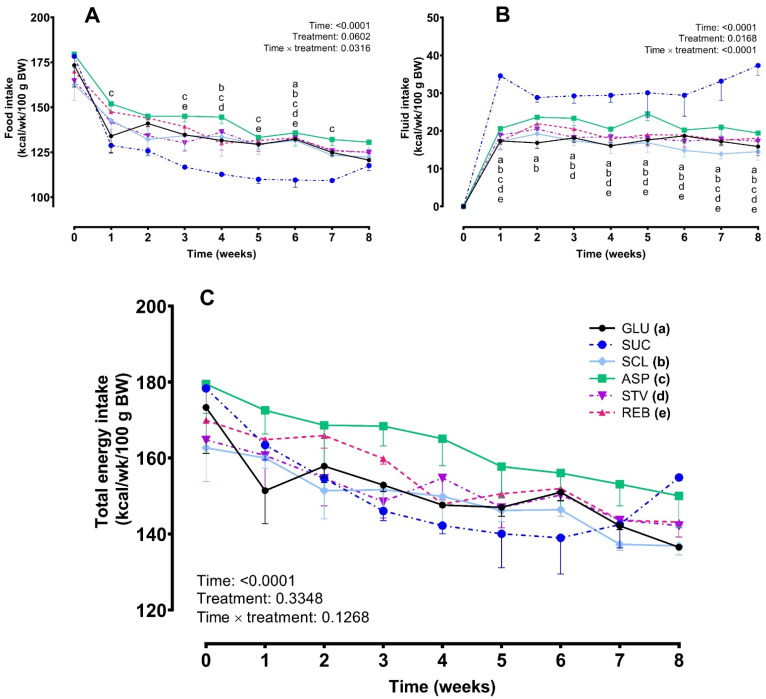
Effects of NNS on food intake (**A**), fluid intake (**B**) and total energy intake (**C**) in the ND-treated group throughout 8 weeks of treatment (phase 2). Data are expressed as mean ± SEM. (*n* = 6–8 animals per group). Two-way RM ANOVA followed by Tukey’s post hoc test was used. Different lowercase letters in each time point indicate significant differences between the sucrose group (SUC) and other groups: (a) GLU, glucose; (b) SCL, sucralose; (c) ASP, aspartame; (d) STV, stevia; (e) REB, reb A (*p* < 0.05).

Data on energy intake from food analyzed by two-way RM ANOVA showed a significant difference in the interaction between time and treatments (*p* = 0.0316) and in the time effect (*p* < 0.0001) but only a trend in the treatment effect (*p* = 0.0602). These differences could be attributed to the lower food consumption in the SUC group in comparison with the other treatments (Figure 3A). The SUC group exhibited a temporal increased fluid consumption compared to the other groups. Analysis of data revealed significant treatment effect (*F* = 7.076, *p* = 0.0168), a significant interaction time × treatment (*F* = 3.760, *p* < 0.0001) and time factor (*F* = 161.7, *p* < 0.0001) (Figure 3B). The effect of the treatments on the total energy intake was similar among groups (*F* = 1.430, *p* = 0.3348) and no significant interaction between time and treatment was observed (*F* = 1.410, *p* = 0.1268) (Figure 3C).

**Figure 4:**
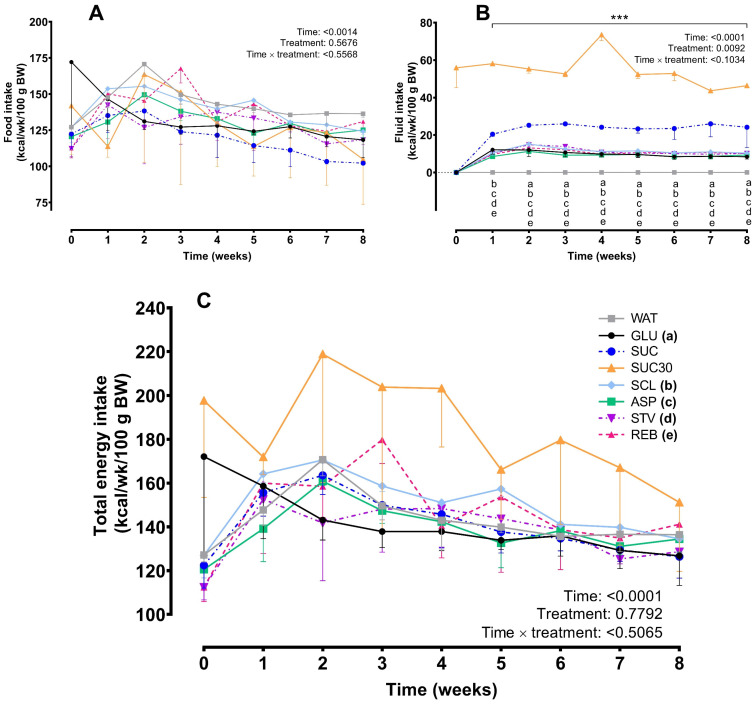
Effects of NNS on food intake (**A**), fluid intake (**B**), and total energy intake (**C**) in the HFD group throughout 8 weeks of treatment (phase 2). Data are expressed as mean ± SEM. (*n* = 6–8 animals per group). Two-way RM ANOVA followed by Tukey’s post hoc test was used. Asterisks indicate significant differences in the 30% sucrose-treated group (SUC30) vs. each other group at *** *p* < 0.0001. Different lowercase letters in each time point indicate significant differences in the sucrose group (SUC) vs. each other group: (a) GLU, glucose; (b) SCL, sucralose; (c) ASP, aspartame; (d) STV, stevia; (e) REB, reb A (*p* < 0.05). Fluid in the water-treated group (WAT) did not contribute calories.

In the HFD-fed rats, no differences were observed between NNS and GLU (control), moreover, in contrast with the ND results, no reduction of the weekly food intake was observed after SUC consumption. Data analysis showed no significant differences among treatments (*F* = 0.3806, *p* = 0.8897) or in the interaction time × treatment (*F* = 0.6175, *p* = 0.9665) (Figure 4A). Regarding fluid intake, data analysis showed differences between treatments (*F* = 132.1, *p* < 0.0001, for treatment effect; *F* = 3.713, *p* < 0.0001 for the interaction time × treatment). As expected, rats on SUC30 consumed higher calories from fluids than any other treatment (*p* < 0.0001) and the fluid in the WAT-treated group did not contribute any calories. In addition, similar to outcomes from the ND group, in the HFD, an increased fluid consumption was observed in the SUC group from the week 1 until week 8 when compared to the other groups (Figure 4B). On the other hand, the total weekly energy intake profile was not different among treatments (Figure 4C) (*F* = 1.678, *p* = 0.2416 for treatment; *F* = 0.5768, *p* = 0.9815 for the interaction time × treatment).

**Figure 5:**
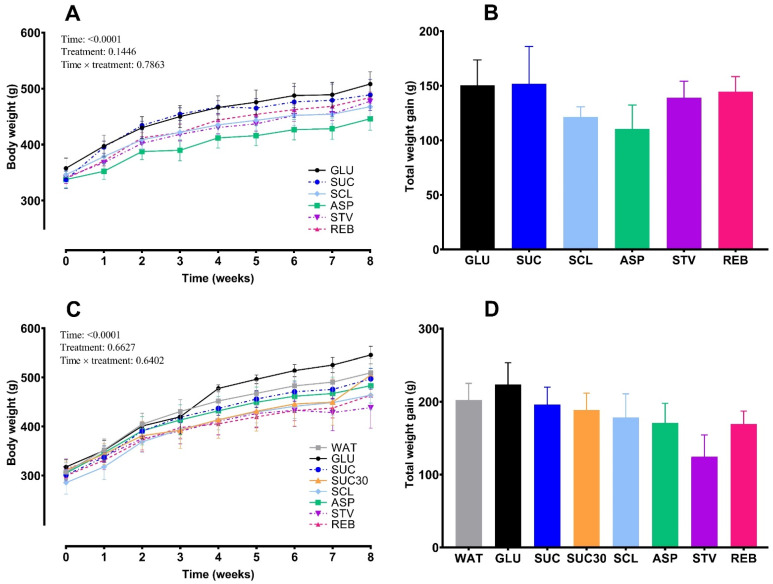
Effects of NNS on weekly body weight throughout 8 weeks of treatment and total weight gain in the ND group (**A**,**B**) and in the HFD group (**C**,**D**) (phase 2). Data are expressed as mean ± SEM. (*n* = 6–8 animals per group). No significant differences among groups were observed after ANOVA on body weight evolution or total weight gain in ND and HFD. WAT, water; GLU, glucose; SUC, sucrose; SUC30, 30% sucrose; SCL, sucralose; ASP, aspartame; STV, stevia; REB, reb A.

In the ND-fed rats, there were no significant differences among groups in weekly body weight evolution throughout the NNS treatments (*F* = 0.8079, *p* = 0.1446 for treatment, *F* = 0.8117, *p* = 0.7863 for the interaction between time and treatment) (Figure 5A). None of the data on total weight gain showed differences among groups (*F* = 0.5993, *p* = 0.7006) (Figure 5B). Likewise, in HFD, treatments did not modify weight gain (*F* = 0.5302, *p* = 0.8079 for treatment; *F* = 0.8666, *p* = 0.7409 for the interaction between time and treatment) (Figure 5C). Moreover, no statistical significance was observed in the total weight gain among the groups on HFD (*F* = 1.235, *p* = 0.3018).

#### 3.2.2. Effects of NNS on Glycemic Response

**Figure 6:**
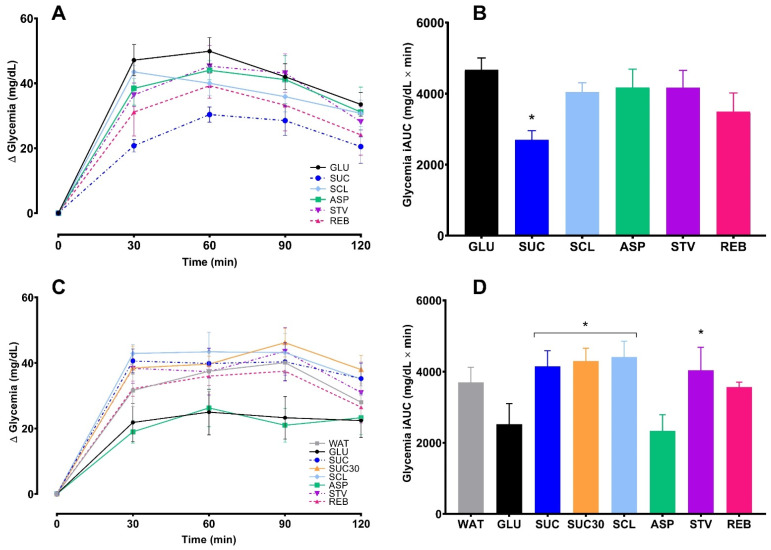
Effects of NNS on glycemic response expressed as change from baseline (∆ glycemia) and incremental area under curves (iAUC), during an oral starch tolerance test (OSTT) over 120 min performed after 12 h of fasting at the end of interventions in ND (**A**,**B**) and HFD-treated group (**C**,**D**). Data are expressed as mean ± SEM. (*n* = 6–8 per group). One-way ANOVA and Dunnett’s post hoc test showed a reduced glycemia iAUC in SUC respect to GLU in the ND group (* *p* < 0.05) and in HFD, an increased glycemia iAUC in SCL, STV, SUC and SUC30 compared to GLU (* *p* < 0.05). WAT, water; GLU, glucose; SUC, sucrose; SUC30, 30% sucrose; SCL, sucralose; ASP, aspartame; STV, stevia; REB, reb A.

**Figure 7:**
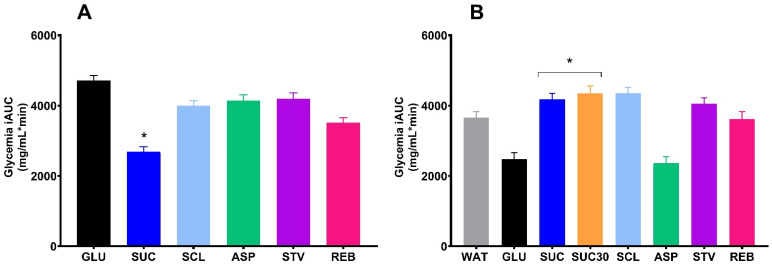
Effects of NNS on glycemia iAUC after the interventions adjusted by the baseline glycemia iAUC values in ND (**A**) and HFD-fed rats (**B**). Data are expressed as mean ± SEM. (*n* = 6–8 per group). No significant differences of NNS were found in either group after ANCOVA analysis. In ND, SUC vs. GLU * *p* < 0.05. In HFD, SUC and SUC30 vs. GLU * *p* < 0.05. WAT, water; GLU, glucose; SUC, sucrose; SUC30, 30% sucrose; SCL, sucralose; ASP, aspartame; STV, stevia; REB, reb A.

From the final OSTT data, the ND group showed no effects of NNS on glycemic profile, however, the SUC glycemia iAUC value was lower in comparison with GLU glycemia iAUC (*p* = 0.001) (Figure 6A,B) and this effect remained even after further testing using the ANCOVA analysis (Figure 7A). In the HFD group, SUC, SUC30, SCL and STV exhibited higher iAUC values respect to GLU (*p* < 0.05) (Figure 6C,D). However, when ANCOVA analysis was performed using the final glycemia AUC as the primary outcome, and the baseline glycemia AUC as a covariate, the NNS effects were removed, even though, SUC and SUC30 groups remained elevated compared to GLU (*p* < 0.05) (Figure 7B). The glycemic profiles before and after the interventions for each sweetener are provided in Supplementary Material, Figures S2 and S3.

#### 3.2.3. Effects of NNS on Fasting Biochemical Parameters

In the ND-fed rats, no effects of SCL, STV or REB were observed on fasting glucose, cholesterol, HDL-cholesterol, insulin or HOMA-IR, only ASP treatment induced an increased total cholesterol level compared to GLU (*p* = 0.0010). Neither in the HFD-fed rats, these effects were observed, however, a significant increase in glucose concentration was observed after SUC30 treatment respect to GLU (*p* = 0.0264), and unexpectedly, the WAT group also showed a significant increase in glucose concentrations compared to GLU (*p* = 0.0283). The HOMA-IR was higher in the HFD group than DN, but not statistical significance was reached (*p* = 0.14). Data are shown in Supplementary Material, Table S1.

## 4. Discussion

In the present study, we evaluated the chronic effects of moderate doses of sucralose, aspartame, stevia or reb A on energy intake, body weight and postprandial glycemia in healthy and with altered glycemic response rats. Findings from the first phase in HCD-treated rats showed a higher weekly energy intake dynamic, especially in the last 4 weeks compared to the ND group. However, the body weight evolution was lower than the ND group and was accompanied by an altered glycemic response after the 8-week treatment period.

Our findings agree with previous studies in which rats on HCD for a few weeks developed glucose intolerance independent of the obesity per se [29]. Some proposed explanatory mechanisms could be the modification in the early insulin response and the high rate of fatty acid oxidation. In fact, the hepatic insulin resistance can appear as early as 1 week after sucrose feeding, while muscle insulin resistance develops until week 2 [30]. The weight loss observed in HCD in respect to the ND group may be partially explained by the lower food consumption (constituted by 13.2% protein) observed in this study. This dietary deficiency can decrease muscle mass by increasing thermogenesis in adipose tissue [31], and induce an increased insulin resistance which is known to reduce muscle protein synthesis [32]. Our findings agree with previous studies where no weight gain has been observed after HCD [33,34].

In the second phase, NNS exposure showed no effects on energy intake from food, fluid and total energy intake in both ND and HFD-treated rats when compared to the control group (GLU), SUC, or WAT during the 8-week treatment period. However, the SUC provoked a reduction in food consumption that was accompanied by an increase in fluid intake in the ND group. These animals compensated for decreases in calories from food in such a way that the total energy intake evolution did not differ among groups. Similar findings have been observed previously and confirm the hypothesis that animals adjust for calories consumed on one occasion by reducing their caloric intake on subsequent opportunities to eat [35,36]. In the same way HFD-treated rats showed a similar increase in fluid intake dynamics after SUC and SUC30; however, in this case, no compensation for food intake was observed. This finding could be a consequence of the profound metabolic alterations induced by the unbalanced diet. Nevertheless, due to the high variability observed in the SUC30 data, and similarly to the ND-fed rats, differences in the total energy intake dynamics were not observed among groups.

In this study, no modifications were observed in body weight evolution or total weight gain after NNS compared to the control group (GLU), in both ND and HFD-fed rats. These results are consistent with those of Bissonnette et al. [37], who reported no effects of stevia and saccharin on weight gain after 6-week treatment period when compared to nonsweetened control. In contrast, Feijó et al. [38] reported that 0.4% aspartame or 0.3% saccharin provided as drinking water to Wistar rats increased BW unrelated to caloric intake when compared to 15% sucrose-fed rats. However, in the latter study, the high NNS doses were provided to healthy animals with restricted physical activity, five days a week for a longer period of 12 weeks. The administered dose was approximately 6.4 times greater than the ADI for aspartame, considering the informed fluid consumption (20 mL of yogurt/d) and the average BW (250 g) of the rats.

In this study, the glycemic responses evaluation was performed through an OSTT administering rapidly digestible starch instead of pure glucose, considering that this substance is not usually consumed by humans, while starch is the most important macronutrient found in everyday foods [24]. Moreover, rapidly digestible starch has been used by our group in previous studies providing quick rises in postprandial glycemia and functioning to analyze glycemic responses [24,39,40]. In this line, some studies have mentioned a lower variability when using starch products instead of pure glucose [34]. When analyzing the NNS effects based on the OSTT at the end of the study, no effects of NNS were observed in the ND-fed rats, and only a reduced glycemia iAUC was appreciated after sucrose. In contrast, in the HFD group, two NNS (SCL and STV) were shown to induce an increased glycemic response. Additionally, SUC and SUC30, also provoked a higher postprandial glycemia profile. These findings agree with other authors reporting an impaired glucose tolerance in rodents after NNS [14,26]. However, in these and other previous studies the statistical analysis only included the glycemia temporal changes from the final OGTT performed at the end of the intervention period without considering a baseline tolerance test data. Here, a baseline OSTT data (baseline glycemia iAUC) was included for adjusting the dependent variable (final glycemia iAUC) through an ANCOVA analysis. After this, no effects of NNS were observed in healthy or with altered glucose tolerance rats. Nevertheless, the differences between GLU and SUC in both groups remained. Our results are consistent with those of Glendinning et al. [41] who reported no effects of sucralose, saccharin or Ace K on glucose tolerance in mice exposed to treatments for 4 weeks. These authors designed a well-controlled study with various OGTTs performed throughout the experimental period and using an appropriate sample size, however, no other NNS types as aspartame or stevia were investigated, and only a standardized diet was administered. In contrast Suez et al. [14] informed that NNS exposure induced glucose intolerance in mice fed regular chow or high-fat diet, however the lack of baseline test could have influenced the results.

The effect of NNS on fasting biochemical parameters was not consistent. ASP induced an increased total cholesterol, but this was not observed in the animals fed an unbalanced diet (HFD). In fact, no effects of other NNS were observed on fasting glycemia, total cholesterol, triglycerides, HDL-cholesterol, insulin or HOMA-IR.

In the present study, commercially available versions of sucralose, aspartame and stevia were used since these are the most common products consumed by the general population and more likely to provide a more accurate representation of the possible physiological effects on humans. Moreover, the administered doses were adjusted by daily fluid consumption and BW to be equivalent to the ADI.

This study has several strengths. Both natural and artificial NNS effects were investigated. Commercial forms of SCL, ASP and STV were used in order to simulate popular consumption. A rat model of diet-induced metabolic dysregulation was used expecting an exacerbation of the glucose intolerance status after NNS consumption. Realistic and moderate NNS doses were provided. Glucose and sucrose-treated rats or water-fed rats served as comparators. A suitable sample size was used. A baseline OSTT was included to discard intra-subjects’ variability. However, some limitations are also present. For instance, not determining body fat composition or other biochemical parameters like leptin, GLP-1, or PYY which could have provided explanations to the metabolic changes related to the appetite control.

## 5. Conclusions

In summary, we have shown that aspartame, sucralose, stevia, and reb A administered at doses equivalent to ADI for 8 weeks did not cause modifications in caloric intake, weight gain, glycemic response or fasting biochemical parameters in healthy and with altered glycemic response rats. Moreover, the results did not differ between artificial and natural NNS. These findings provide further evidence suggesting that at the recommended doses, these substances do not alter feeding behavior, body weight or glycemic tolerance.

## Data Availability

The data presented in this study are available on request from the corresponding author.

## Supplementary Materials

The following are available online at https://www.mdpi.com/article/10.3390/foods10050958/s1, Figure S1: Variation in the NNS consumption throughout the 8 weeks of experimental period in ND (left) and HFD-fed rats (right) (phase 2), Figure S2: Glycemic profiles before and after the interventions in ND-fed rats (A–F) (phase 2), Figure S3: Glycemic profiles before and after the interventions in HFD-fed rats (A–H) (phase 2). Table S1: Effects of NNS on fasting biochemical parameters.

## References

[1] Lazzarino G.P., Acutain M.F., Canesini G., Andreoli M.F., Ramos J.G. (2019). Cafeteria diet induces progressive changes in hypothalamic mechanisms involved in food intake control at different feeding periods in female rats. Mol. Cell. Endocrinol..

[2] Das A., Chakraborty R., Mérillon J.-M., Ramawat K.G. (2018). An introduction to sweeteners. Sweeteners: Pharmacology, Biotechnology, and Applications.

[3] Dunford E., Taillie L., Miles D., Eyles H., Tolentino-Mayo L., Ng S. (2018). Non-nutritive sweeteners in the packaged food supply—an assessment across 4 countries. Nutrients.

[4] Martyn D., Darch M., Roberts A., Lee H.Y., Yaqiong Tian T., Kaburagi N., Belmar P. (2018). Low-/no-calorie sweeteners: A review of global intakes. Nutrients.

[5] Romo-Romo A., Aguilar-Salinas C.A., Gómez-Díaz R., Brito-Córdova G.X., Gómez-Velasco D.V., López-Rocha M.J., Almeda-Valdes P. (2017). Non-nutritive sweeteners: Evidence on their association with metabolic diseases and potential effects on glucose metabolism and appetite. Rev. Investig. Clin..

[6] Laviada H., Molina Segui F. (2017). Posición de la Sociedad Mexicana de Nutrición y Endocrinología sobre los edulcorantes no calóricos. Rev. Mex. Endocrinol. Metab. Nutr..

[7] Pearlman M., Obert J., Casey L. (2017). The association between artificial sweeteners and obesity. Curr. Gastroenterol. Rep..

[8] Olivier-Van Stichelen S., Rother K.I., Hanover J.A. (2019). Maternal exposure to non-nutritive sweeteners impacts progeny’s metabolism and microbiome. Front. Microbiol..

[9] Pepino M.Y., Bourne C. (2011). Non-nutritive sweeteners, energy balance, and glucose homeostasis. Curr. Opin. Clin. Nutr. Metab. Care.

[10] Fantino M., Fantino A., Matray M., Mistretta F. (2018). Beverages containing low energy sweeteners do not differ from water in their effects on appetite, energy intake and food choices in healthy, non-obese French adults. Appetite.

[11] Ford H.E., Peters V., Martin N.M., Sleeth M.L., Ghatei M.A., Frost G.S., Bloom S.R. (2011). Effects of oral ingestion of sucralose on gut hormone response and appetite in healthy normal-weight subjects. Eur. J. Clin. Nutr..

[12] Pinto D.E., Foletto K.C., Nunes R.B., Lago P.D., Bertoluci M.C. (2017). Long-term intake of saccharin decreases post-absortive energy expenditure at rest and is associated to greater weight gain relative to sucrose in wistar rats. Nutr. Metab..

[13] Pepino M.Y., Tiemann C.D., Patterson B.W., Wice B.M., Klein S. (2013). Sucralose affects glycemic and hormonal responses to an oral glucose load. Diabetes Care.

[14] Suez J., Korem T., Zeevi D., Zilberman-Schapira G., Thaiss C.A., Maza O., Israeli D., Zmora N., Gilad S., Weinberger A. (2014). Artificial sweeteners induce glucose intolerance by altering the gut microbiota. Nature.

[15] Romo-Romo A., Aguilar-Salinas C.A., Brito-Córdova G.X., Gómez-Díaz R.A., Almeda-Valdes P. (2018). Sucralose decreases insulin sensitivity in healthy subjects: A randomized controlled trial. Am. J. Clin. Nutr..

[16] Higgins K.A., Considine R.V., Mattes R.D. (2018). Aspartame consumption for 12 weeks does not affect glycemia, appetite, or body weight of healthy, lean adults in a randomized controlled trial. J. Nutr..

[17] Bonnet F., Tavenard A., Esvan M., Laviolle B., Viltard M., Lepicard E.M., Lainé F. (2018). Consumption of a carbonated beverage with high-intensity sweeteners has no effect on insulin sensitivity and secretion in nondiabetic adults. J. Nutr..

[18] Andrejić B.M., Mijatović V.M., Samojlik I.N., Horvat O.J., Ćalasan J.D., Đolai M.A. (2013). The influence of chronic intake of saccharin on rat hepatic and pancreatic function and morphology: Gender differences. Bosn. J. Basic Med. Sci..

[19] Lobach A.R., Roberts A., Rowland I.R. (2019). Assessing the in vivo data on low/no-calorie sweeteners and the gut microbiota. Food. Chem. Toxicol..

[20] Mattes R.D., Popkin B.M. (2009). Nonnutritive sweetener consumption in humans: Effects on appetite and food intake and their putative mechanisms. Am. J. Clin. Nutr..

[21] FDA Additional Information about High-Intensity Sweeteners Permitted for Use in Food in the United States. https://www.fda.gov/food/food-additives-petitions/additional-information-about-high-intensity-sweeteners-permitted-use-food-united-states.

[22] Sengupta P. (2013). The laboratory rat: Relating its Age with human’s. Int. J. Prev. Med..

[23] Wolever T.M.S., Vuksan V., Palmason C. (1996). Less variation of postprandial blood glucose after starchy test meals than oral glucose. Nutr. Res..

[24] Ble-Castillo J.L., Aparicio-Trapala M.A., Juárez-Rojop I.E., Torres-Lopez J.E., Mendez J.D., Aguilar-Mariscal H., Olvera-Hernández V., Palma-Cordova L.C., Diaz-Zagoya J.C. (2012). Differential effects of high-carbohydrate and high-fat diet composition on metabolic control and insulin resistance in normal rats. Int. J. Environ. Res. Public Health.

[25] Mooradian A.D., Smith M., Tokuda M. (2017). The role of artificial and natural sweeteners in reducing the consumption of table sugar: A narrative review. Clin. Nutr. ESPEN.

[26] Rosales-Gomez C.A., Martinez-Carrillo B.E., Resendiz-Albor A.A., Ramirez-Duran N., Valdes-Ramos R., Mondragon-Velasquez T., Escoto-Herrera J.A. (2018). Chronic consumption of sweeteners and its effect on glycaemia, cytokines, hormones, and lymphocytes of GALT in CD1 mice. BioMed Res. Int..

[27] Barrios-Correa A.A., Estrada J.A., Martel C., Olivier M., Lopez-Santiago R., Contreras I. (2018). Chronic intake of commercial sweeteners induces changes in feeding behavior and signaling pathways related to the control of appetite in BALB/c mice. BioMed Res. Int..

[28] Matthews D.R., Hosker J.P., Rudenski A.S., Naylor B.A., Treacher D.F., Turner R.C. (1985). Homeostasis model assessment: Insulin resistance and β-cell function from fasting plasma glucose and insulin concentrations in man. Diabetologia.

[29] la Fleur S.E., Luijendijk M.C.M., van Rozen A.J., Kalsbeek A., Adan R.A.H. (2010). A free-choice high-fat high-sugar diet induces glucose intolerance and insulin unresponsiveness to a glucose load not explained by obesity. Int. J. Obes..

[30] Pagliassotti M.J., Prach P.A., Koppenhafer T.A., Pan D.A. (1996). Changes in insulin action, triglycerides, and lipid composition during sucrose feeding in rats. Am. J. Physiol..

[31] Ceolín P., Franca S.A.D., Froelich M., Santos M.P.D., Pereira M.P., Queiroz T.S., Silva F.H.S.D., Lisboa P.C., Andrade C.M.B., Baviera A.M. (2019). A low-protein, high carbohydrate diet induces increase in serum adiponectin and preserves glucose homeostasis in rats. An. Acad. Bras. Cienc..

[32] Gatineau E., Savary-Auzeloux I., Migné C., Polakof S., Dardevet D., Mosoni L. (2015). Chronic intake of sucrose accelerates sarcopenia in older male rats through alterations in insulin sensitivity and muscle protein synthesis. J. Nutr..

[33] Burgeiro A., Cerqueira M.G., Varela-Rodriguez B.M., Nunes S., Neto P., Pereira F.C., Reis F., Carvalho E. (2017). Glucose and lipid dysmetabolism in a rat model of prediabetes induced by a high-sucrose diet. Nutrients.

[34] Gomez-Crisostomo N.P., De la Cruz-Hernandez E.N., Mendez Mendez E.R., Hernandez-Landero M.F., Camacho Lievano J.U., Martinez-Abundis E. (2020). Differential effect of high-fat, high-sucrose and combined high-fat/high-sucrose diets consumption on fat accumulation, serum leptin and cardiac hypertrophy in rats. Arch. Physiol. Biochem..

[35] Mazlan N., Horgan G., Stubbs R.J. (2006). Energy density and weight of food effect short-term caloric compensation in men. Physiol. Behav..

[36] Rowland N.E., Nasrallah N., Robertson K.L. (2005). Accurate caloric compensation in rats for electively consumed ethanol–beer or ethanol–polycose mixtures. Pharmacol. Biochem. Behav..

[37] Bissonnette D.J., List S., Knoblich P., Hadley M. (2017). The effect of nonnutritive sweeteners added to a liquid diet on volume and caloric intake and weight gain in rats. Obesity.

[38] Feijó F.d.M., Ballard C.R., Foletto K.C., Batista B.A.M., Neves A.M., Ribeiro M.F.M., Bertoluci M.C. (2013). Saccharin and aspartame, compared with sucrose, induce greater weight gain in adult Wistar rats, at similar total caloric intake levels. Appetite.

[39] Garcia-Vazquez C., Ble-Castillo J.L., Arias-Cordova Y., Cordova-Uscanga R., Tovilla-Zarate C.A., Juarez-Rojop I.E., Olvera-Hernandez V., Alvarez-Villagomez C.S., Nolasco-Coleman A.M., Diaz-Zagoya J.C. (2019). Effects of Resistant Starch Ingestion on Postprandial Lipemia and Subjective Appetite in Overweight or Obese Subjects. Int. J. Environ. Res. Public Health.

[40] Ble-Castillo J.L., Juárez-Rojop I.E., Tovilla-Zárate C.A., García-Vázquez C., Servin-Cruz M.Z., Rodríguez-Hernández A., Araiza-Saldaña C.I., Nolasco-Coleman A.M., Díaz-Zagoya J.C. (2017). Acute consumption of resistant starch reduces food intake but has no effect on appetite ratings in healthy subjects. Nutrients.

[41] Glendinning J.I., Hart S., Lee H., Maleh J., Ortiz G., Ryu Y.S., Sanchez A., Shelling S., Williams N. (2020). Low-calorie sweeteners cause only limited metabolic effects in mice. Am. J. Physiol. Regul. Integr. Comp. Physiol..

