# Supplementary material for: Effects of Non-Nutritive Sweeteners on Energy Intake, Body Weight and Postprandial Glycemia in Healthy and with Altered Glycemic Response Rats
Source: Foods. 2021 Apr 28;10(5):958. doi: 10.3390/foods10050958 (PMC8146401; doi:10.3390/foods10050958)
Supplement: Supplementary file 1 [file foods-10-00958-s001.zip › foods-1159765-supplementary/Figures S1, S2 and S3 captions.pdf]

Figure S1. Variation in the NNS consumption throughout the 8 weeks of experimental period in ND (left) and HFD-fed rats (right) (phase 2). (**A**, **B**) SCL, sucralose. (**C**, **D**) ASP, aspartame. (**E**, **F**) STV, Stevia. (**G**, **H**) REB, reb A. The dotted horizontal line observed in each figure represents the ideal dose (equivalent to 1 ADI) for each NNS. Data are expressed as mean ± standard deviation. n = 6–8 animals per group. ADI, acceptable daily intake

Figure S2. Glycemic profiles before and after the interventions in ND-fed rats (**A**–**F**) (phase 2). Data are expressed as mean ± SEM. n = 6–8 animals per group. Two-way repeated measures ANOVA and Sidak's test, *$p < 0.05$ after *vs* before treatments. GLU, glucose; SUC, sucrose; SCL, sucralose; ASP, aspartame; STV, stevia; REB, reb A.

Figure S3. Glycemic profiles before and after the interventions in HFD-fed rats (**A–H**) (phase 2). Data are expressed as mean ± SEM. n = 6–8 animals per group. Two-way repeated measures ANOVA and Sidak's test, *$p < 0.05$ after *vs* before treatments. WAT, water; GLU, glucose; SUC, sucrose; SUC30, 30% sucrose; SCL, sucralose; ASP, aspartame; STV, stevia; REB, reb A.
