# Supplementary material for: Effects of Non-Nutritive Sweeteners on Energy Intake, Body Weight and Postprandial Glycemia in Healthy and with Altered Glycemic Response Rats
Source: Foods. 2021 Apr 28;10(5):958. doi: 10.3390/foods10050958 (PMC8146401; doi:10.3390/foods10050958)
Supplement: Supplementary file 1 [file foods-10-00958-s001.zip › foods-1159765-supplementary/Table S1.pdf]

Table S1. Effects of NNS on fasting biochemical parameters

	HFD							
	**WAT**	**GLU**	**SUC**	**SUC30**	**SCL**	**ASP**	**STV**	**REB**
Glycemia (mg/dL)	163.9±18.53*	111.4±9.76	125.6±6.70	166.5±11.80*	126.3±14.88	145.9±10.74	128.3±14.54	123.3±8.46
Insulin (μU/mL)	150.4±28.59	56.51±12.87	86.44±25.36	154.3±28.59	65.60±36.13	115.0±25.93	145.3±36.22	28.13±19.19
HOMA-IR (mg/dL×μU/mL)	53.26±8.94	16.95±4.71	28.80±9.92	63.70±11.62	29.31±20.46	43.78±11.28	52.36±13.09	8.43±5.78
Total cholesterol (mg/dL)	51.38±3.55	51±3.73	65.63±4.57	64.83±4.90	53±3.56	59.43±3.09	55.63±4.65	55.5±4.84
Triglycerides (mg/dL)	55.17±6	48.14±3.49	62.63±5.81	51.60	52.57±5.50	54.14±6.34	52.29±5.83	52.83±2.96
HDL-cholesterol (mg/dL)	19.83±1.27	18.51±0.81	24.3±2.08	23.52±1.03	19.65±1.04	22.49±2.62	20.01±1.46	20.47±1.03
	ND							
		**GLU**	**SUC**		**SCL**	**ASP**	**STV**	**REB**
Glycemia (mg/dL)		123.3±6.88	127.9±9.93		120.4±12.28	115±4.98	120.4±8.69	137.9±12.42
Insulin (μU/mL)		79.35±24.29	72.55±20.42		102.3±27.76	43.80±8.09	66.10±34.37	76.33±29.93
HOMA-IR (mg/dL×μU/mL)		26.51±8.97	25.62±8.21		30.84±7.97	11.48±1.97	23.95±13.96	29.53±13.43
Total cholesterol (mg/dL)		51.38±4.95	49.63±3.44		64.5±3.81	76.43±5.27*	57.29±5.19	65±2.95
Triglycerides (mg/dL)		38.63±3.02	41.25±4.22		45±1.72	55.71±9.06	56.29±3.98	48.25±4.21
HDL-cholesterol (mg/dL)		21.01±1.44	20.86±0.67		24.89±1.30	23.43±2.45	21.73±1.66	25.61±0.73

Values are mean ± SEM. HFD, high fat diet; ND, normal diet; WAT, water; GLU, glucose; SUC, sucrose; SUC30, 30% sucrose; SCL, sucralose; ASP, aspartame; STV, stevia; REB, reb A. HOMA-IR, homeostatic model assessment. One-way ANOVA followed by Dunnet's post hoc test was used. Asterisks indicate significant differences in the glucose-treated group (GLU) vs. each other group at $p < 0.05$.
